# Experience in Implementing Colonization Screening in a Multidisciplinary County Clinical Hospital in Romania

**DOI:** 10.3390/microorganisms13040775

**Published:** 2025-03-28

**Authors:** Victoria Birlutiu, Rares-Mircea Birlutiu, Razvan Ene, Dana Rusu

**Affiliations:** 1 Faculty of Medicine, Lucian Blaga University of Sibiu, Str. Lucian Blaga, Nr. 2A, 550169 Sibiu, Romania; victoria.birlutiu@ulbsibiu.ro; 2 Infectious Diseases Department, County Clinical Emergency Hospital, Bvd Corneliu Coposu, Nr. 2–4, 550245 Sibiu, Romania; 3 Faculty of Medicine, University of Medicine and Pharmacy “Carol Davila”, 050474 Bucharest, Romania; 4 Clinical Hospital of Orthopedics, Traumatology, and Osteoarticular TB Bucharest, B-dul Ferdinand 35–37, Sector 2, 021382 Bucharest, Romania; 5 Clinical Department No. 14, “Carol Davila” University of Medicine and Pharmacy, 020021 Bucharest, Romania; razvan.ene@umfcd.ro; 6 Healthcare-Associated Infection Prevention Service, County Clinical Emergency Hospital, Bvd Corneliu Coposu, Nr. 2–4, 550245 Sibiu, Romania; dana.rusu@scjus.ro

**Keywords:** colonization screening, multidrug-resistant organisms, infection control, screening protocols, antibiotic resistance

## Abstract

Skin microbiota plays a crucial role in host defense. Disruptions in this balance can lead to systemic infections. Colonization by pathogenic microorganisms significantly increases the risk of symptomatic infections, prolongs hospital stays and increases healthcare costs. In Romania, the widespread misuse of antibiotics in the community further complicates the management of bacterial resistance, emphasizing the need for proactive measures. Our institution implemented a comprehensive multi-body-site colonization screening protocol starting from January 2024 until June 2024. The screening targeted high-risk patients, including those in ICUs, Oncology, and Hematology Clinics, and individuals with prior hospitalizations, antibiotic use, or medical devices. This study aimed to investigate the prevalence of colonization by multidrug-resistant organisms upon hospital admission and assess the changes in colonization rates during hospitalization. Samples from nasal, axillary, inguinal, and rectal swabs were processed on specialized chromogenic media to detect multidrug-resistant organisms such as methicillin-resistant *S. aureus* (MRSA), ESBL-producing *Enterobacterales*, and carbapenemase-producing bacteria. During the first two trimesters of the year 2024, 1522 patients aged 14 to 99 years underwent multi-body-site colonization screening at the County Clinical Emergency Hospital Sibiu, Romania. A total of 18,993 samples were analyzed, yielding a diverse range of bacterial isolates. The most common results were *Escherichia coli*-ESBL-negative (3584 cases, 18.9%) and the KESC bacteria group (*Klebsiella* spp., *Enterobacter* spp., *Serratia* spp., and *Citrobacter* spp.)-MDR-negative (3435 cases, 18.1%). Conversely, positive results were less frequent, with *Acinetobacter baumannii*-MDR-positive results in 62 cases (0.3%), *E. coli*-ESBL-positive results in 342 cases (1.8%), and KESC group-MDR-positive results in 491 cases (2.6%). Other notable findings included *Enterococcus faecium*-VRE-positive (157 cases, 0.8%) and MRSA-positive nasal swabs (141 cases, 0.7%). Rare isolates included *Enterococcus faecalis*-VRE-positive (4 cases, 0.0%) and Proteeae group-MDR-positive (33 cases, 0.2%). Negative screening for MRSA was prevalent across nasal (1850 cases, 9.7%), inguinal (742 cases, 3.9%), and axillary swabs (1124 cases, 5.9%), with substantially lower positive rates. The diversity of swab types and their distribution across various clinics and departments underscores the broad diagnostic approaches and patient-care strategies adopted during the study. These findings highlight the need for tailored infection prevention strategies and continuous surveillance to mitigate the spread of multidrug-resistant organisms and enhance patient safety across diverse clinical environments.

## 1. Introduction

Skin hosts a vast array of bacteria, fungi, and viruses that collectively form the skin microbiota. These microorganisms are essential for host defense, contributing to protection against invasive pathogens, modulation of immune responses, and the breakdown of natural substances. As the largest organ of the human body, the skin is colonized by beneficial microbes, which function as a physical barrier to pathogen entry. However, disruptions of this barrier or imbalances between commensal and pathogenic organisms can lead to skin pathology or even systemic disease. Interactions between members of the microbiota shape the resident microbial community and inhibit the colonization of pathogenic bacteria through a process known as colonization resistance. Notably, under certain conditions, microorganisms typically considered symbiotic can acquire pathogenic characteristics, underscoring the complexity of host-microbe interactions on the skin [1].

Patients colonized with pathogenic microorganisms face a significantly elevated risk of developing symptomatic infections, which are closely linked to prolonged hospitalizations and increased healthcare expenditures. Various targeted interventions aimed at reducing infection rates have been extensively examined, and the potential advantages of implementing universal patient screening protocols in hospital settings are currently under consideration at the policy level in numerous countries worldwide [2,3].

In Romania, the implementation of patient colonization screening upon admission was recently introduced at the end of 2023. As such, there is no national data on the prevalence of colonization among hospitalized patients. Local epidemiology is undoubtedly distinct, with factors such as patient comorbidities, recent hospitalizations, and antibiotic use in the general population potentially influencing the regional differences. Understanding colonization patterns, particularly when initiating decontamination measures, can significantly reduce the risk of infections, especially in surgical services, where surgical site infections remain the most common complication in both Europe and the USA [4]. In Germany, it is estimated that 20–30% of the population is colonized with *Staphylococcus aureus* on the skin [5], of which less than 1% is MRSA. However, the resistance rate increases among patients with prolonged hospital stay [6].

Colonization with vancomycin-resistant enterococci (VRE), even in European countries with relatively low levels of multidrug resistance such as Switzerland [7], is closely monitored following an outbreak involving over 500 patients who were either colonized or infected with VRE [8]. These cases predominantly occurred in patients who were previously hospitalized in another healthcare facility. In Italy, an increase in VRE colonization among hematology patients has also been observed, from 2.6% in 2018 per 1000 patient days to 4.6% per 1000 patient days in 2022 [9]. The issue of VRE has been recognized in the USA since 1989, necessitating the implementation of surveillance and control measures, early identification of colonization or infection with VRE, case isolation, and restrictions on vancomycin administration [10].

Colonization with carbapenem-resistant *Klebsiella pneumoniae* (CR-KP), particularly rectal colonization and involvement of multiple sites, is associated with an increased risk of systemic infections, as highlighted by Gianella’s study in Italy [11]. Colonization with CR-KP during hospitalization is also associated with an increased risk of infection compared to patients already colonized at admission, highlighting the need for colonization monitoring during hospital stays exceeding seven days [12].

The issue of antibiotic resistance in Romania, particularly the widespread use of antibacterial treatments without etiological testing, poses significant challenges in identifying effective first-line therapies for hospitalized patients. This trend undermines efforts to combat bacterial resistance and complicates decisions regarding empiric antibiotic therapy. This is especially problematic for cases without recent hospitalizations or antibiotic use within the last three months, where there is a higher risk that initial antibiotic treatments may prove ineffective.

Given these circumstances, implementing a comprehensive screening protocol for hospitalized patients is essential. This rigorous approach aimed to proactively identify and manage antibiotic-resistant colonization or infection in our patient population, safeguarding both individual and public health within our institution.

This study aimed to investigate the prevalence of colonization by multidrug-resistant organisms upon hospital admission and assess the changes in colonization rates during hospitalization. In addition, we aimed to describe the patterns of MDRO colonization across multiple body sites in hospitalized patients.

## 2. Materials and Methods

### 2.1. Study Design

This single-center observational retrospective cohort study was conducted at the County Clinical Emergency Hospital Sibiu, Romania. The study received ethical approval from the hospital’s ethics committee (file number 21433/28-08-2024) and underwent review by the institutional management board, also securing its approval. The authors accessed the electronic medical records and data from 1 July 2024 to 31 July 2024. The authors had access to information that could identify individual participants during data collection.

### 2.2. Study Population

We retrospectively selected all consecutive patients who were hospitalized between 1 January 2024 and 30 June 2024. Patient information was extracted directly from laboratory records using a standardized electronic form, ensuring that comprehensive data were available for all the enrolled patients. This information underwent a rigorous evaluation to verify its accuracy and reliability, facilitating the acquisition of robust data for our analysis. The data were meticulously reviewed and cross-verified by two authors to ensure its accuracy, reliability, and consistency.

A standardized multi-body-site colonization screening system was implemented and used to assess all patients who underwent multi-body-site colonization screening during the study period. Our employed multi-body-site colonization screening strategy included the screening of all patients admitted to the Anesthesia and Intensive Care Unit Clinics from any medical or surgical department, especially if they were expected to be admitted for at least 24 h, patients admitted to Oncology and Hematology Clinics, those with hospitalizations in the past year, individuals flagged by the hospital’s information system as colonized or infected with multidrug-resistant organisms in the past three months, residents of long-term care facilities, patients transferred from other hospitals regardless of their previous length of stay, those who had received antibiotics within the last year, patients with medical devices, and those with stomas (colostomy, nephrostomy, or gastrostomy). Additionally, screening was extended to the roommates of patients with MDRO colonization or infection. For ICU cases, contacts were defined as patients occupying adjacent rooms during the same period as a confirmed MDRO-positive case. Screening for these individuals included nasal and rectal swabs to detect multidrug-resistant Gram-negative bacteria and vancomycin-resistant *Enterococci* (VRE), as well as inguinal or axillary swabs.

All patients scheduled for elective surgeries were screened for methicillin-resistant *Staphylococcus aureus*. At the Orthopedics and Traumatology Clinic, nasal and inguinal swabs were collected, while nasal and axillary swabs were also obtained for scheduled vascular and thoracic surgeries.

### 2.3. Laboratory Studies

For the multi-body-site colonization screening program initiated in our hospital, sterile swab kits were used to collect samples for culture and sensitivity (C and S) testing of aerobic bacteria from multiple body sites, including nasal swabs, axillary swabs, inguinal swabs, and rectal swabs. This action was performed by nurses following hospital infection control protocols. Nasal swabs were inoculated onto chromogenic plates specific to MRSA, whereas inguinal and axillary swabs were also plated for MRSA detection. Rectal swabs were processed on chromogenic media for Gram-negative bacteria to screen for extended-spectrum beta-lactamase (ESBL)-producing organisms, carbapenemase-producing bacteria, and multidrug-resistant *Acinetobacter baumannii* (AB MDR). A range of chromogenic culture media from bioMérieux (Marcy-l’Étoile, France) and CHROMagar™ (Paris, France) were used to facilitate the targeted isolation and identification of multidrug-resistant organisms. Specifically, CHROMID^®^ (Paris, France) media included CHROMID MRSA/CHROMID *S. aureus* bi-plate for detecting *S. aureus* and MRSA, CHROMID VRE for vancomycin-resistant enterococci (VRE), and CHROMID Carba for carbapenemase-producing organisms. Additionally, CHROMagar™ media was utilized with CHROMagar™ *Acinetobacter* for *Acinetobacter* species detection, CHROMagar™ ESBL for identifying Gram-negative bacteria producing extended-spectrum beta-lactamase (ESBL), CHROMagar™ KPC and CHROMagar™ mSuperCARBA™ for carbapenem-resistant Enterobacteriaceae (CRE), and CHROMagar™ MRSA and CHROMagar™ VRE for isolating MRSA and vancomycin-resistant *Enterococcus faecalis* and *Enterococcus faecium*, and also Colorex™ ESBL (CHROMagar™ (Paris, France)) to identify *Proteus* spp., *Providencia* spp., and *Morganella* spp. This comprehensive selection of detection media enabled the effective screening of multidrug-resistant organisms across multiple body sites, contributing valuable insights into colonization patterns and infection risks.

### 2.4. Statistical Analyses

Statistical analyses were performed using the IBM SPSS Statistics version 29 (Chicago, IL, USA). Continuous variables were summarized using univariate descriptive statistics, reporting counts, and percentages where applicable. The Mann–Whitney test was used to compare skewed distributions of continuous variables, whereas categorical variables were assessed using the Chi-Square, Fisher’s exact, or Cramer’s V tests, as appropriate.

## 3. Results

During the first two trimesters of the year 2024, 1522 patients underwent multi-body-site colonization screening in the County Clinical Emergency Hospital Sibiu, Romania, with 43.8% (*n* = 666) screened in the first trimester of the year and 56.2% (*n* = 856) in the second trimester of the year.

Our study included patients from various medical departments, as it is highlighted in Table 1. The most significant proportion of patients who were screened were admitted to the first Anesthesia and Intensive Care Unit Clinic, comprising 27.6% (*n* = 420) of the total enrolled patients. This was followed by the first Internal Medicine Clinic, which accounted for 20.1% (*n* = 306) of the patients, and a second Internal Medicine Clinic, which included 14.5% (*n* = 221).

A crosstabulation between the distribution of patients that underwent multi-body-site colonization screening across various medical departments over the first two trimesters of the year 2024 has also been performed and is reported in Appendix A Table A1. The first Anesthesia and Intensive Care Unit Clinic recorded the highest number of patients who underwent screening for colonization, with 205 patients in the first trimester of the year and 215 in the second trimester of the year, accounting for 420 patients. The performed crosstabulation analysis showed a statistically significant link between the trimesters of the year of multi-body-site bacterial colonization screening and the hospital department (χ^2^(27) = 92.567, *p* < 0.001), which was further confirmed by the likelihood ratio test (χ^2^(27) = 107.556, *p* < 0.001). While the correlation between these factors was relatively weak, it was still statistically significant, as reflected in Pearson’s R (R = 0.117, *p* < 0.001) and Spearman’s rank correlation (ρ = 0.122, *p* < 0.001).

The ages of the patients enrolled in the study ranged from 14 to 99 years, with a mean age of 68.05 years (SD: 14.49). In the first trimester of the year, the mean age was 68.44 years (95% CI: 67.37–69.51; SD: 14.09; range of 18–96 years), and in the second trimester of the year, the mean age was 67.75 years (95% CI: 66.76–68.74; SD: 14.79; range 14–99 years). Both trimesters of the year demonstrated a tendency towards an older patient demographic, with similar central tendency measures and dispersion, although the second trimester of the year showed slightly greater age variability. Of the 1522 patients enrolled, 820 (53.9%) were male and 702 (46.1%) female, with a slight male predominance. In the first trimester of the year, 363 males and 303 females were enrolled, compared to 457 males and 399 females in the second. This consistent gender distribution highlights a slight increase in enrollment for both genders during the second trimester of the year.

The crosstabulation between age and gender by trimester of the year revealed no statistically significant association in our study.

**Figure 1:**
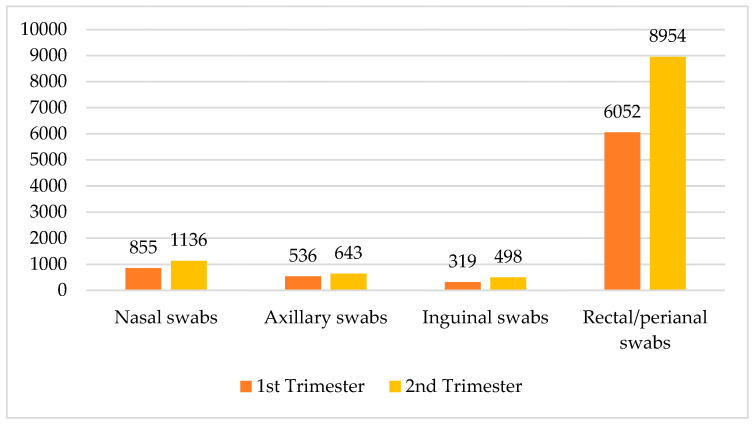
Distribution of swabs by trimester of the year and type.

Across all departments, a total of 18,993 samples were collected, with 11,231 in the second trimester of the year and 7762 in the first trimester of the year. The data demonstrate a consistent increase in sample collection in the second trimester of the year. The crosstabulation data detail the collection of various sample types across the two trimesters of the year, categorized into nasal swabs, axillary swabs, inguinal swabs, and rectal/perianal swabs. In the second trimester of the year, a total of 11,231 samples were collected, comprising 1136 nasal swabs, 643 axillary swabs, 498 inguinal swabs, and 8954 rectal/perianal swabs. Comparatively, the first trimester of the year accounted for 7762 samples, with 855 nasal swabs, 536 axillary swabs, 319 inguinal swabs, and 6052 rectal/perianal swabs. Overall, across both trimesters of the year, the total number of swabs was 1991 nasal swabs, 1179 axillary swabs, 817 inguinal swabs, and 15,006 rectal/perianal swabs. These data revealed a notable increase in sample collection from the first to the second trimester of the year, particularly in rectal/perianal swabs, which represented the majority of samples in both periods (see Figure 1).

The crosstabulation data are presented in Appendix A Table A2, which highlights a detailed account of the multi-body-site colonization screening samples collected across multiple medical departments, categorized by the type of swabs and trimester of the year. The types of samples used include nasal swabs, axillary swabs, inguinal swabs, and rectal/perianal swabs.

### 3.1. Screening Results

**Figure A1:**
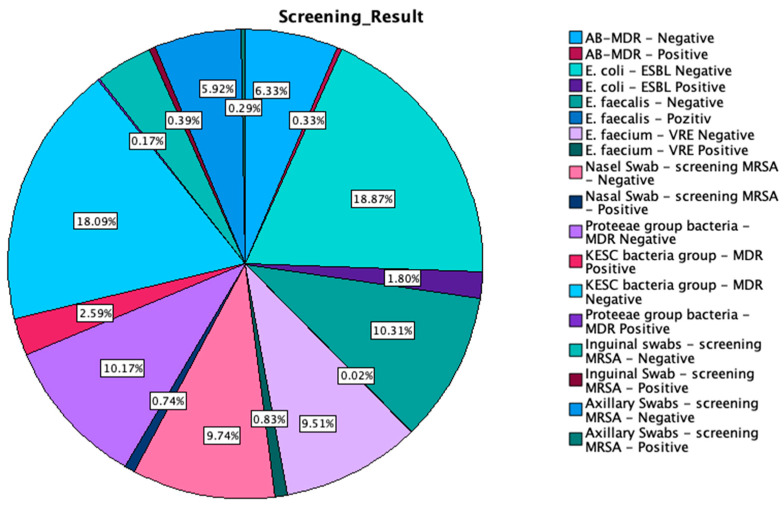
Graphical representation of the multi-body-site colonization screening results.

The multi-body-site colonization screening results of our study encompass a wide range of bacterial isolates across the 18,993 samples, each categorized by their specific outcome. The most prevalent results included *E. coli*-ESBL (extended-spectrum beta-lactamase)-producing-negative (3584 cases, 18.9%) and KESC (*Klebsiella* spp., *Enterobacter* spp., *Serratia* spp., and *Citrobacter* spp.) bacteria group-MDR (multidrug-resistant)-negative (3435 cases, 18.1%). Notably, AB-MDR (*Acinetobacter baumanii* multidrug-resistant)-negative was reported in 1202 cases (6.3%), while the Proteeae group bacteria-MDR-negative accounted for 1931 cases (10.2%). In contrast, positive results were relatively infrequent, with AB-MDR-positive isolated in 62 cases (0.3%), *E. coli*-ESBL-positive in 342 cases (1.8%), and KESC bacteria group-MDR-positive in 491 cases (2.6%). Additionally, *Enterococcus faecium*-VRE (vancomycin-resistant *Enterococcus*)-positive and nasal swab-screening MRSA-positive were found in 157 (0.8%) and 141 (0.7%) cases, respectively. Among the least isolated strains were *E. faecalis*-positive and Proteeae group bacteria-MDR-positive, with only four (0.0%) and 33 (0.2%) cases, respectively. Negative screenings for MRSA were notably common across nasal (1850 cases, 9.7%), inguinal (742 cases, 3.9%), and axillary swabs (1124 cases, 5.9%), with positive results being substantially lower in these categories. A summary of the multi-body-site colonization screening results is highlighted in Table 2 and Appendix A Figure A1.

**Figure 2:**
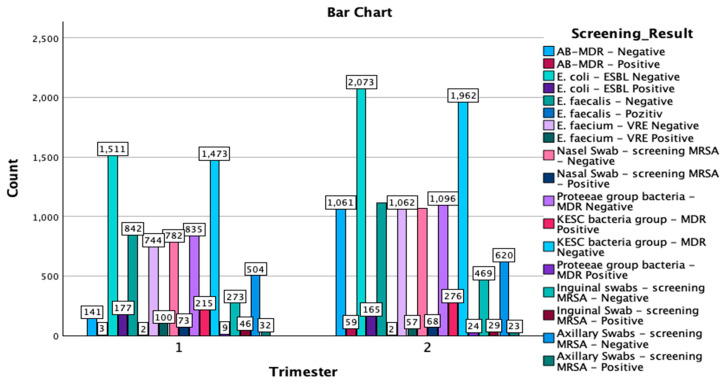
Graphical representation of the screening results by trimester of the year and isolated type of strains. MDR—multidrug resistant; ESBL—extended-spectrum beta-lactamase; KESC—*Klebsiella* spp., *Enterobacter* spp., *Serratia* spp., and *Citrobacter* spp.; AB-MDR—*Acinetobacter baumanii* multidrug resistant; VRE—vancomycin-resistant *Enterococcus*; MRSA—methicillin-resistant *Staphylococcus aureus*.

The crosstabulation of the multi-body-site colonization screening results by trimester of the year provides a comprehensive overview of the distribution of various bacterial strains and antibiotic resistance patterns detected in the samples. A total of 18,993 samples were analyzed, with 7762 samples collected in the first trimester of the year and 11,231 in the second trimester of the year (Figure 2).

Methicillin-resistant *Staphylococcus aureus* screening results:

A total of 55 positive cases were detected through axillary swabs, with 32 occurring in the first trimester of the year and 23 in the second, while 1124 samples were negative, with 504 in the first trimester of the year and 620 in the second trimester of the year. Inguinal swabs identified 75 positive cases, 46 of which were reported in the first trimester of the year and 29 in the second, while 742 samples tested negative, with 273 from the first trimester of the year and 469 from the second trimester of the year. Nasal swabs revealed 141 positive cases out of 1991 screenings, with 73 positive cases in the first trimester of the year and 68 in the second trimester of the year. The remaining 1850 samples were negative, with 782 from the first trimester of the year and 1068 from the second trimester of the year.

### 3.2. Other Key Findings

In the KESC bacteria group, the most substantial number of cases were in the MDR-negative category, with 3435 total negative samples—1473 cases from the first trimester of the year and 1962 cases from the second. Additionally, 491 cases were MDR-positive, with 215 cases identified in the first trimester of the year and 276 cases in the second trimester of the year. For the Proteeae group, only 33 positive MDR cases were reported, with an increase from 9 cases in the first trimester of the year to 24 cases in the second trimester of the year. Most samples in this group were MDR-negative, totaling 1931 cases, with 835 cases in the first trimester of the year and 1096 cases in the second. In the *Enterococcus faecium*—VRE category, there were 157 positive cases, with a higher number in the first trimester of the year (100 cases) compared to the second trimester of the year (57 cases), alongside 1806 negative cases overall. Lastly, for *Escherichia coli*-ESBL, 3584 samples were ESBL-negative (1511 in the first trimester of the year and 2073 in the second trimester of the year), while 342 samples were ESBL-positive, with 177 cases in the first trimester of the year and 165 cases in the second.

The crosstabulation analysis of screening results by trimester of the year reveals important distributions of positive and negative cases for various bacterial screenings across the two study periods. *E. coli*-ESBL-negative showed the highest prevalence, with 1511 cases in the first trimester of the year and 2073 cases in the second trimester of the year, resulting in a total of 3584 cases. Similarly, the KESC bacteria group-MDR-negative displayed substantial numbers, with 1473 cases in the first trimester of the year and 1962 in the second trimester of the year, amounting to 3435 cases overall. In contrast, AB-MDR-positive cases were relatively infrequent, with only three cases reported in the first trimester of the year and 59 in the second trimester of the year, for a total of 62 cases. For methicillin-resistant *Staphylococcus aureus* screenings, nasal swab-negative cases were predominant, with 782 in the first trimester of the year and 1068 in the second (totaling 1850), while there were fewer positive cases (73 in the first and 68 in the second, totaling 141). A similar trend was observed for axillary and inguinal swabs also, with negative results vastly outnumbering positive results. The Chi-Square tests indicated a significant association between the trimester of the year and the distribution of screening results (χ^2^(17) = 567.752, *p* < 0.001), with the likelihood ratio test confirming this result (χ^2^(17) = 652.078, *p* < 0.001). The Linear-by-Linear Association test also showed significant findings (χ^2^(1) = 90.814, *p* < 0.001), suggesting a consistent pattern across the trimester of the years. The symmetric measures, including Pearson’s R (−0.069) and Spearman’s correlation (−0.071), both with *p* < 0.001, indicate a weak negative association between the trimester of the year and the screening results.

**Figure 3:**
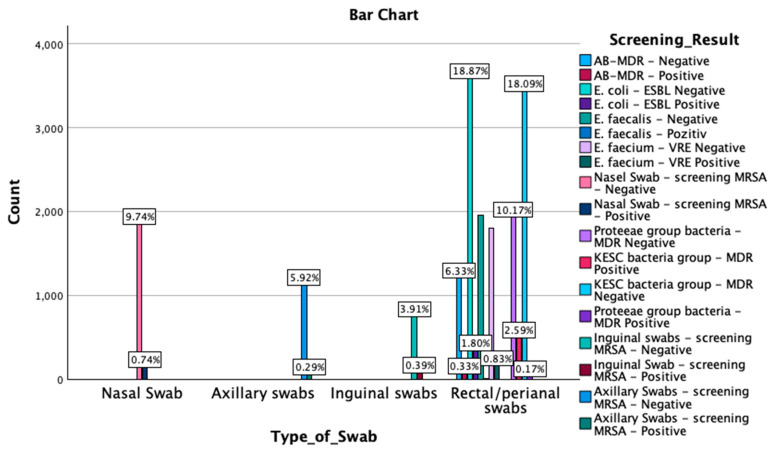
Graphical representation of the screening results by type of swab. MDR—multidrug resistant; ESBL—extended-spectrum beta-lactamase; KESC—*Klebsiella* spp., *Enterobacter* spp., *Serratia* spp., and *Citrobacter* spp.; AB-MDR—*Acinetobacter baumanii* multidrug resistant; VRE—vancomycin-resistant *Enterococcus*; MRSA—methicillin-resistant *Staphylococcus aureus*.

A crosstabulation between screening results by type of swab was also performed. The Chi-Square test results revealed a significant association between the type of swab and the screening outcomes (χ^2^(51) = 56,979.000, *p* < 0.001). Similarly, the likelihood ratio indicated a strong association (χ^2^(51) = 27,747.278, *p* < 0.001), as did the Linear-by-Linear Association test (χ^2^(1) = 2069.462, *p* < 0.001). Symmetric measures, including Pearson’s R (−0.330) and Spearman’s correlation (−0.391), demonstrated significant negative correlations (*p* < 0.001). These findings suggest that the type of swab used is strongly associated with the detection outcomes, with the negative correlations indicating an inverse relationship between the swab type and the screening results. An overview of these findings is reported in Figure 3 and Appendix A Table A3.

### 3.3. Detailed Interpretation of the Screening Results by Department

Table A4 represents an overview of the screening results reported by each department.

Across various hospital departments, the multi-body-site colonization screening revealed significant findings. The first Anesthesia and Intensive Care Unit Clinic reported the highest number of positive screenings, with 6261 cases, including notable detections of *E. coli*-ESBL-negative (1210 cases), KESC-MDR-negative (1126 cases), and several AB-MDR-positive cases (15 cases). The first Internal Medicine Clinic followed, with 3095 positive results, highlighting *E. coli*-ESBL-negative (586 cases) and KESC-MDR-negative (606 cases), along with a few AB-MDR-positive (5 cases) and *E. coli*-ESBL-positive cases (58 cases). The second Internal Medicine Clinic recorded 2520 positive screenings, with substantial findings in *E. coli*-ESBL-negative (472 cases) and KESC-MDR-negative (469 cases), as well as some *E. coli*-ESBL-positive (48 cases) and AB-MDR-positive cases (1 case).

In the Infectious Diseases Clinic, 1729 positive results were noted, with significant detections of *E. coli*-ESBL-negative (311 cases) and KESC-MDR-negative (317 cases), alongside a few AB-MDR-positive cases (8 cases). The Anesthesia and Intensive Care Unit Dedicated to Neurosurgical, Plastic Surgery, and Other Surgical Departments reported 1653 positive results, particularly *E. coli*-ESBL-negative (312 cases), KESC-MDR-negative (252 cases), and several AB-MDR-positive (26 cases) and KESC-MDR-positive cases (92 cases).

The Cardiology Intensive Care Department had 581 positive screenings, predominantly detecting *E. coli*-ESBL-negative (111 cases) and AB-MDR-negative (49 cases), with some KESC-MDR-positive cases (13 cases). The Rheumatology Clinic recorded 205 positive cases, primarily *E. coli*-ESBL-negative (36 cases) and Proteeae group-MDR-negative (21 cases), along with a few KESC-MDR-positive cases. The Medical Rehabilitation Clinic—Neurological Department reported 445 positive cultures, including *E. coli*-ESBL-negative (88 cases) and Proteeae group-MDR-negative (45 cases), with a few AB-MDR-positive cases. Lastly, the Medical Rehabilitation Clinic—Orthopedics and Traumatology Department identified significant isolates, particularly *E. coli*-ESBL-negative (51 cases) and Proteeae group-MDR-negative (27 cases).

**Figure A2:**
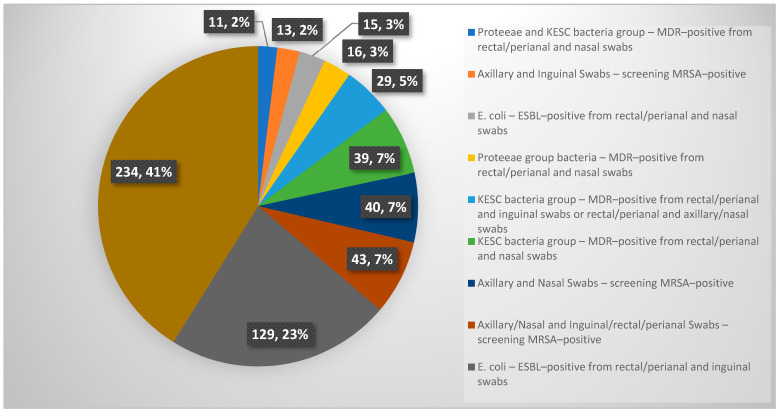
Graphical representation of simultaneous at least two positive results from multi-body-site colonization screening.

Among the patients screened during the study, 569 patients exhibited simultaneously at least two positive results from multi-body-site colonization screening. Notably, 11 patients showed co-colonization with Proteeae and KESC bacteria groups-MDR-positive from rectal/perianal and nasal swabs. Similarly, 13 patients tested MRSA-positive from axillary and inguinal swabs, while 15 cases involved *E. coli*-ESBL-positive findings from rectal/perianal and nasal swabs. Cases of Proteeae group bacteria-MDR-positive colonization from rectal/perianal and nasal swabs were observed in 16 patients, whereas 29 patients demonstrated KESC bacteria group MDR positivity across combinations of rectal/perianal and inguinal or axillary/nasal swabs. Additionally, 39 patients showed MDR-positive KESC bacteria from rectal/perianal and nasal swabs. Screening for MRSA also revealed 40 positive cases from axillary and nasal swabs, with an additional 43 cases identified from combined axillary/nasal and inguinal/rectal/perianal swabs. The most significant findings were observed in *E. coli*-ESBL-positive cases from rectal/perianal and inguinal swabs, which accounted for 129 patients. Strikingly, 234 patients exhibited complex patterns of colonization involving more than two bacterial species and multiple screening sites, underscoring the need for tailored infection control strategies in such scenarios. A graphical representation of these data is provided in Appendix A Figure A2.

Out of the 1522 patients who underwent multi-site colonization screening at the County Clinical Emergency Hospital Sibiu during the first two trimesters of the year 2024, 934 had hospital stays exceeding seven days or required transfer between departments. In these instances, a subsequent multi-site colonization screening was performed. Notably, in 114 cases, discrepancies were observed between the colonization results at admission and those obtained during the subsequent screening, accounting for 150 positive swabs. The multi-site colonization screening conducted across various anatomical locations similar to the one conducted at the time of admission, including rectal/perianal, inguinal, axillary, and nasal sites, revealed significant findings regarding the prevalence of multidrug-resistant organisms. Among the rectal/perianal swabs, there were 2 cases of *Acinetobacter baumanii* conversion from MDR-negative to MDR-positive, while 4 cases were observed in the inguinal area. *Escherichia coli* colonization showed a marked increase, with 34 cases turning from negative to positive in the rectal/perianal region. *Enterococcus faecium*, with respect to vancomycin resistance (VRE), had 8 positive cases in rectal/perianal swabs. Furthermore, the KESC bacteria group exhibited a significant rise in MDR-positive cases, with 50 recorded at the rectal/perianal site. Similarly, the Proteeae group bacteria demonstrated 8 positive cases. For MRSA screenings, the axillary swabs identified 12 positive cases, the inguinal swabs identified 10 positive cases, and the nasal swabs revealed 22 positive cases.

## 4. Discussion

The decision to implement comprehensive hospital multi-site colonization screening has become imperative because of the increasing antibiotic resistance among major Gram-positive and Gram-negative pathogens, a challenge that has become even more pressing in the post-pandemic period. This multi-site colonization screening program offers significant benefits, including the early identification of antibiotic-resistant profiles in patients upon admission, isolation of individuals carrying multidrug-resistant organisms to prevent their spread within the hospital environment, and optimization of antibiotic therapy based on local resistance patterns.

Active screening protocols are not consistently implemented across Europe, and there is considerable variation in testing sites; for example, some protocols focus on the axillary or inguinal regions for colonization assessment. Additionally, certain practitioners tend to interpret colonization as an active infection, leading to unnecessary antibiotic use in cases that do not require curative intervention.

Studies have reported that approximately 20% of patients colonized with carbapenem-resistant *Enterobacteriaceae* (CRE) progress to developing infections. This risk is significantly influenced by factors such as the number of colonization sites, the presence of a central venous catheter, and prior exposure to vancomycin preceding colonization [13]. Other studies have reported an even higher infection rate of 25% among patients colonized with CRE, which is associated with a negative impact on 60-day survival rates [14]. Patients colonized with multidrug-resistant Gram-negative bacteria (MDR-GNB) present a significant risk of environmental contamination, particularly on surfaces and medical equipment. This risk increases during invasive procedures or the use of portable medical devices [15]. Other studies, such as that by Richter et al. [16], have identified specific risk factors associated with the development of carbapenem resistance among Gram-negative bacilli (excluding *Pseudomonas aeruginosa*, which is intrinsically resistant to ertapenem). These factors include underlying renal disease, prior hospitalization in another healthcare facility, recent use of antibiotics effective against MRSA or carbapenems within the preceding 30 days, and need for mechanical ventilation [16].

Colonization with carbapenem-resistant *Klebsiella* spp. and the presence of OXA-type carbapenemases are significant risk factors for the development of infections in these patients [17]. Rectal colonization with CRE can occur upon patient admission, with reported prevalence rates ranging from 8.8% to 25.5% [11,18,19]. The prevalence of rectal colonization with CRE is influenced by prior antibiotic therapies as well as patient comorbidities. Additional risk factors for the development of infections among colonized patients include colonization at multiple sites, polymicrobial etiology, antibiotic use within the past 90 days, concurrent respiratory infections, albumin administration, catheterization, and other invasive procedures [14,17]. Among the antibiotics administered prior to colonization with carbapenem-resistant *Enterobacterales*, the most commonly identified are fluoroquinolones, third- and fourth-generation cephalosporins, carbapenems, and antipseudomonal penicillins [13,20].

Multidrug-resistant (MDR) bacterial infections have emerged as the most significant healthcare challenge of the past decade, a situation that is further exacerbated in the post-pandemic era. Addressing this issue is a top priority for the World Health Organization (WHO), emphasizing the urgent need to develop new, effective therapies against MDR pathogens. Efforts must focus on limiting infections and identifying therapeutic solutions for critical infections caused by carbapenem-resistant *Pseudomonas aeruginosa* (CR *P. aeruginosa*), carbapenem-resistant *Acinetobacter baumannii* (CR *A. baumannii*), and carbapenem-resistant or extended-spectrum beta-lactamase-producing Enterobacterales (ESBL-producing CRE) [21,22]. The second tier of high-priority pathogens includes vancomycin-resistant *Enterococcus faecium* (VRE), MRSA, vancomycin-intermediate and vancomycin-resistant *S. aureus* (VISA/VRSA), *Helicobacter pylori* resistant to clarithromycin, fluoroquinolone-resistant *Campylobacter* and *Salmonella*, and *Neisseria gonorrhoeae* resistant to cefalosporins and fluoroquinolones. Addressing these pathogens requires a concerted global effort to mitigate the growing threat of antibiotic resistance [21,22]. Although we have not previously published specific data on this particular subject from our hospital, there are two published manuscripts from our center that provide relevant insights into multidrug-resistant organisms [23,24]. One such study reported data on multidrug-resistant periprosthetic joint infections (PJIs), identifying a total of 48 multidrug-resistant bacterial strains isolated during the study period. These included 14 MRSA and 17 multidrug-resistant strains of Gram-negative aerobic bacilli strains. Interestingly, MRSA and multidrug-resistant Gram-negative bacilli were simultaneously involved in five PJI cases. The study highlighted the following species as accounting for all multidrug-resistant Gram-negative aerobic bacilli isolated: *Pseudomonas* spp. (four strains), *Escherichia coli* (five strains), *Acinetobacter* spp. (two strains), *Enterobacter cloacae* complex (three strains) and *Ralstonia pickettii* (three strains). Notably, 10 out of the 17 multidrug-resistant Gram-negative aerobic bacilli strains were extended-spectrum β-lactamase (ESBL)-producing Enterobacterales. Despite these findings, no statistically significant linear trend indicating an increase or decrease in the prevalence of multidrug-resistant bacteria was observed during the study period [24].

In our study, the prevalence of MDR colonization at admission was relatively low, with rates of 0.3% for MDR *Acinetobacter baumannii* (AB-MDR-positive), 1.8% for *Escherichia coli* (ESBL-positive), and 2.6% for the KESC bacterial group (MDR-positive). *Enterococcus faecium* (VRE) and MRSA were identified in 0.8% and 0.7% of cases, respectively.

MRSA was isolated from 55 axillary swabs, 141 nasal swabs, and 75 inguinal swabs. Among rectal swabs, MDR *Proteeae* group bacteria were identified in 33 samples, while the KESC group (MDR-positive) was detected in 491 samples. *Enterococcus faecium* (VRE) was found in 157 rectal swabs, and *Enterococcus faecalis* (VRE) was isolated from 4 rectal swabs. Additionally, 342 rectal swabs tested positive for ESBL-positive *E. coli*, while AB-MDR was present in 62 rectal samples.

These findings provide valuable insights into the colonization patterns of MDR organisms upon hospital admission and underscore the importance of systematic screening protocols to guide infection prevention strategies.

As expected, the majority of positive tests originated from the Anesthesia and Intensive Care Unit, which is dedicated to the Neurosurgical, Plastic Surgery, and Other Surgical Departments. Among the 1653 samples collected, the following MDR-positive organisms were identified: 26 were AB-MDR, 32 were ESBL-positive *E. coli*, 26 were *E. faecium* VRE, 21 were MRSA from nasal swabs, 2 were axillary swabs, 30 were inguinal swabs, 92 were KESC-MDR-positive, and 4 were strains of *Proteeae*. A significant proportion of patients admitted to the Infectious Diseases Department also showed MDR colonization, primarily those transferred from the Intensive Care Unit. Out of the 1729 samples, the following results were noted: 8 AB-MDR, 49 ESBL-positive *E. coli*, 1 *E. faecalis* VRE, 15 *E. faecium* VRE, 12 MRSA from nasal swabs, 14 from inguinal swabs, 1 from axillary swabs, 43 KESC-MDR, and 4 MDR *Proteeae*.

The Internal Medicine departments played a significant role in screening and collecting 3095 and 2520 samples, respectively. Conversion from sensitive isolates to MDR-positive strains was observed in 114 patients, including *A. baumannii*, *E. coli*, *E. faecium*, *Proteeae*, and predominantly the KESC group (50 cases) and MRSA (44 cases). These conversions were associated with the use of third-generation cephalosporins, fluoroquinolones, or antipseudomonal penicillins.

These findings highlight the urgent need to consider stricter antibiotic stewardship measures to limit MDR colonization, particularly in high-risk patient populations [25,26]. The use of albumin to prevent infections in patients colonized with CRE has been associated with enhanced antimicrobial activity of vasostatin-I [27].

Decolonization strategies for patients colonized with MRSA have been shown to effectively reduce the risk of morbidity and mortality associated with subsequent infections. This approach plays a crucial role in preventing the transition from colonization to active infection, particularly in vulnerable patient populations, thereby improving clinical outcomes and enhancing overall patient safety [28].

### The Role of Host and Environmental Flora in Hospital-Acquired Infections

Hospitalized patients introduce their own microbiota, which, under specific clinical conditions, can contribute to infection risk. For instance, *Pseudomonas aeruginosa* commonly colonizes tracheostomy- and ventilator-dependent patients, whereas multidrug-resistant *Enterobacteriaceae* may be present in the gastrointestinal tract of neurologically impaired patients with neurogenic bladder and recurrent urinary tract infections. These resident organisms can disseminate into the hospital environment, where they may be acquired directly by other patients or indirectly via healthcare workers’ (HCWs) hands. The hospital setting itself serves as a reservoir for resistant pathogens such as methicillin-resistant *Staphylococcus aureus*, vancomycin-resistant *Enterococcus*, multidrug-resistant gram-negative bacteria, and *C. difficile*. Transmission can occur through direct contact with contaminated surfaces, indirect transfer via fomites or HCW hands, or patient-to-patient spread facilitated by HCWs. Hospital surfaces act as both reservoirs and vehicles for transmission, harboring pathogens such as MRSA, *Pseudomonas aeruginosa*, *Acinetobacter* spp., and *C. difficile*, with contamination levels influenced by infection site, patient type, and cleaning practices. *C. difficile*, in particular, is challenging to eradicate because of its spore resilience and is frequently detected in patient rooms. The risk of contamination extends to HCW gloves and hands following patient contact or interaction with contaminated surfaces. Furthermore, prior room occupants infected with healthcare-associated pathogens may contribute to cross-contamination, increasing the risk of infection for subsequent patients occupying the same space [29].

While this study provides valuable insights into colonization screening, it has several limitations that should be acknowledged. Conducted as a single-center study at the County Clinical Emergency Hospital Sibiu, the findings may not fully generalize to other institutions with varying patient demographics, healthcare practices, or regional epidemiological trends. The relatively short six-month study period may have missed seasonal or annual variations in colonization patterns, and the cross-sectional design limits the ability to assess long-term trends or outcomes, such as the impact of interventions or progression to infection. The focus on a specific subset of patients, selected based on defined screening criteria, introduces the possibility of selection bias, potentially limiting broader applicability. Detailed hospitalization history, prior antibiotic exposure, morbidity status, and admission diagnoses were not included in the analysis, as these data were not extracted. Furthermore, the study did not assess the progression from colonization to infection or its potential impact on patient outcomes. The two aspects mentioned above were beyond the scope of the current investigation; hence, future studies will incorporate these variables. Additionally, while the use of chromogenic media enabled effective MDRO detection, the lack of molecular typing has restricted deeper insights into the epidemiology and transmission dynamics of the identified organisms. Lastly, resource and data documentation limitations may have influenced both the sensitivity of the detection methods and the consistency of the extracted information. Despite these challenges, the study highlights important aspects of colonization screening, providing a foundation for future multicenter and longitudinal research to address these gaps and enhance infection prevention strategies.

## 5. Conclusions

The diversity of swab types and their distribution across various clinics and departments underscores the broad diagnostic approaches and patient care strategies adopted during the study. This comprehensive screening effort provides valuable insights into the prevalence of multidrug-resistant organisms and other bacterial strains within the study population, offering a critical foundation for evaluating infection control measures and informing clinical management strategies. Our data revealed a marked increase in sample collection during the second trimester of the year, reflecting intensified diagnostic activities. While the prevalence of MRSA, ESBL-producing *E. coli*, and other resistant strains emphasizes the need for vigilant infection control practices, the relatively low number of positive cases in several categories indicates that the majority of the patient population was not colonized with these resistant bacteria. The variability in bacterial prevalence and resistance patterns across departments is noteworthy. High-volume units have demonstrated significant detection rates for various pathogens, underscoring the critical need for robust infection control measures in these settings. These findings highlight the need for tailored infection prevention strategies and continuous surveillance to mitigate the spread of multidrug-resistant organisms and enhance patient safety across diverse clinical environments.

## Figures and Tables

**Table 1:** Patient distribution across different departments.

Department			
	Frequency (*n*=)	Percent	Cumulative Percent
Allergology Department	11	0.7	0.7
1st Anesthesia and Intensive Care Unit Clinic	420	27.6	28.3
Anesthesia and Intensive Care Unit Dedicated to Neurosurgical, Plastic Surgery, and Other Surgical Departments	63	4.1	32.5
Infectious Diseases Clinic	156	10.2	42.7
1st Cardiology Clinic	10	0.7	43.4
2nd Cardiology Clinic	10	0.7	44.0
1st Surgical Clinic—Thoracic Surgery Department	6	0.4	44.4
2nd Surgical Clinic—Vascular Surgery Department	6	0.4	44.8
1st Surgical Clinic	2	0.1	44.9
2nd Surgical Clinic	8	0.5	45.5
Diabetes Clinic	1	0.1	45.5
Gastroenterology Clinic	7	0.5	46.0
Haematology Clinic	25	1.6	47.6
1st Internal Medicine Clinic	306	20.1	67.7
2nd Internal Medicine Clinic	221	14.5	82.3
Neurosurgical Clinic	8	0.5	82.8
Neurology Clinic	23	1.5	84.3
Obstetrics and Gynecology Clinic	1	0.1	84.4
Oncology Clinic	5	0.3	84.7
Orthopedics and Traumatology Clinic	96	6.3	91.0
1st Medical Rehabilitation Clinic	6	0.4	91.4
Medical Rehabilitation Clinic—Neurological Department	29	1.9	93.3
Medical Rehabilitation Clinic—Orthopedics and Traumatology Department	16	1.1	94.3
Rheumatology Clinic	19	1.2	95.6
Cardiology Intensive Care Department	54	3.5	99.1
Urology Clinic	8	0.5	99.7
2nd Anesthesia and Intensive Care Unit Clinic	2	0.1	99.8
Nephrology and Dialysis Clinic	3	0.2	100.0
Total	1522	100.0	

**Table 2:** Isolated strain during the study period.

Screening Result		
	Frequency (*n*=)	Percent (%)
AB-MDR-negative	1202	6.3
AB-MDR-positive	62	0.3
*E. coli*-ESBL-negative	3584	18.9
*E. coli*-ESBL-positive	342	1.8
*E. faecalis*-negative	1959	10.3
*E. faecalis*-positive	4	0
*E. faecium*-VRE-negative	1806	9.5
*E. faecium*-VRE-positive	157	0.8
Nasal swab-screening MRSA-negative	1850	9.7
Nasal swab-screening MRSA-positive	141	0.7
Proteeae group bacteria-MDR-negative	1931	10.2
KESC bacteria group-MDR-positive	491	2.6
KESC bacteria group-MDR-negative	3435	18.1
Proteeae group bacteria-MDR-positive	33	0.2
Inguinal swab-screening MRSA-negative	742	3.9
Inguinal swab-screening MRSA-positive	75	0.4
Axillary swab-screening MRSA-negative	1124	5.9
Axillary swab-screening MRSA-positive	55	0.3
Total	18,993	100.0

MDR—multidrug resistant; ESBL—extended-spectrum beta-lactamase; KESC—*Klebsiella* spp., *Enterobacter* spp., *Serratia* spp., and *Citrobacter* spp.; AB-MDR—*Acinetobacter baumanii* multidrug resistant; VRE—vancomycin-resistant *Enterococcus*; MRSA—methicillin-resistant *Staphylococcus aureus*.

## Data Availability

The original contributions presented in this study are included in the article. Further inquiries can be directed to the corresponding author.

## Appendix A

**Table A1:** Department by Trimester of the Year Crosstabulation.

		Trimester of the Year		Total
		1	2	
Department	Allergology Department	8	3	11
	1st Anesthesia and Intensive Care Unit Clinic	205	215	420
	Anesthesia and Intensive Care Unit Dedicated to Neurosurgical, Plastic Surgery, and Other Surgical Departments	39	24	63
	Infectious Diseases Clinic	65	91	156
	1st Cardiology Clinic	3	7	10
	2nd Cardiology Clinic	6	4	10
	1st Surgical Clinic—Thoracic Surgery Department	3	3	6
	2nd Surgical Clinic—Vascular Surgery Department	2	4	6
	1st Surgical Clinic	1	1	2
	2nd Surgical Clinic	4	4	8
	Diabetes Clinic	1	0	1
	Gastroenterology Clinic	2	5	7
	Haematology Clinic	6	19	25
	1st Internal Medicine Clinic	150	156	306
	2nd Internal Medicine Clinic	85	136	221
	Neurosurgical Clinic	5	3	8
	Neurology Clinic	0	23	23
	Obstetrics and Gynecology Clinic	0	1	1
	Oncology Clinic	3	2	5
	Orthopedics and Traumatology Clinic	42	54	96
	1st Medical Rehabilitation Clinic	1	5	6
	Medical Rehabilitation Clinic—Neurological Department	5	24	29
	Medical Rehabilitation Clinic—Orthopedics and Traumatology Department	3	13	16
	Rheumatology Clinic	14	5	19
	Cardiology Intensive Care Department	9	45	54
	Urology Clinic	4	4	8
	2nd Anesthesia and Intensive Care Unit Clinic	0	2	2
	Nephrology and Dialysis Clinic	0	3	3
Total		666	856	1522

**Table A2:** Type of swabs by Department by Trimester of the Year Crosstabulation.

Department			Type of Swab				Total
			Nasal Swab	Axillary Swabs	Inguinal Swabs	Rectal/Perianal Swabs	
Nephrology and Dialysis Clinic	Trimester of the year	2	3	2	1	24	30
	Total		3	2	1	24	30
2nd Anesthesia and Intensive Care Unit Clinic	Trimester of the year	2	2	2		16	20
	Total		2	2		16	20
Urology Clinic	Trimester of the year	1	4	1	3	28	36
		2	5	4	1	40	50
	Total		9	5	4	68	86
Cardiology Intensive Care Department	Trimester of the year	1	10	6	4	71	91
		2	49	6	43	392	490
	Total		59	12	47	463	581
Rheumatology Clinic	Trimester of the year	1	16	16		123	155
		2	5	5		40	50
	Total		21	21		163	205
Medical Rehabilitation Clinic—Orthopedics and Traumatology Department	Trimester of the year	1	3	1	2	22	28
		2	24	8	16	192	240
	Total		27	9	18	214	268
Medical Rehabilitation Clinic—Neurological Department	Trimester of the year	1	5	0	5	36	46
		2	40	22	19	328	409
	Total		45	22	24	364	455
1st Medical Rehabilitation Clinic	Trimester of the year	1	1	0	1	7	9
		2	6	4	3	48	61
	Total		7	4	4	55	70
Orthopedics and Traumatology Clinic	Trimester of the year	1	44	5	39	242	330
		2	55	7	50	288	400
	Total		99	12	89	530	730
Oncology Clinic	Trimester of the year	1	3	2	1	22	28
		2	2	1	1	16	20
	Total		5	3	2	38	48
Obstetrics and Gynecology Clinic	Trimester of the year	2	1		1	8	10
	Total		1		1	8	10
Neurology Clinic	Trimester of the year	2	26	16	10	208	260
	Total		26	16	10	208	260
Neurosurgical Clinic	Trimester of the year	1	5	3	2	35	45
		2	3	1	2	24	30
	Total		8	4	4	59	75
2nd Internal Medicine Clinic	Trimester of the year	1	101	1	100	728	930
		2	159	0	159	1272	1590
	Total		260	1	259	2000	2520
1st Internal Medicine Clinic	Trimester of the year	1	155	149	6	1111	1674
		2	167	165	2	1340	1421
	Total		322	314	8	2451	3095
Haematology Clinic	Trimester of the year	1	7	7		51	65
		2	21	21		168	210
	Total		28	28		219	275
Gastroenterology Clinic	Trimester of the year	1	2	2		14	18
		2	6	6		48	60
	Total		8	8		62	78
Diabetes Clinic	Trimester of the year	1	1		1	7	9
	Total		1		1	7	9
2nd Surgical Clinic	Trimester of the year	1	4	4		28	36
		2	5	5		40	50
	Total		9	9		68	86
1st Surgical Clinic	Trimester of the year	1	1	1	0	7	9
		2	1	0	1	8	10
	Total		2	1	1	15	19
2nd Surgical Clinic—Vascular Surgery Department	Trimester of the year	1	3	3		21	27
		2	4	4		32	40
	Total		7	7		53	67
1st Surgical Clinic—Thoracic Surgery Department	Trimester of the year	1	3	3		23	29
		2	4	4		32	40
	Total		7	7		55	69
2nd Cardiology Clinic	Trimester of the year	1	6	3	3	42	54
		2	4	3	1	32	40
	Total		10	6	4	74	94
1st Cardiology Clinic	Trimester of the year	1	3	2	1	21	27
		2	7	5	2	56	70
	Total		10	7	3	77	97
Infectious Diseases Clinic	Trimester of the year	1	81	5	76	577	739
		2	99	1	98	792	990
	Total		180	6	174	1369	1729
Anesthesia and Intensive Care Unit Dedicated to Neurosurgical, Plastic Surgery, and Other Surgical Departments	Trimester of the year	1	96	7	69	545	697
		2	95	12	83	766	956
	Total		171	19	152	1311	1653
1st Anesthesia and Intensive Care Unit Clinic	Trimester of the year	2	313	308	5	2234	2860
		1	340	337	4	2720	3401
	Total		653	645	9	4954	6261
Allergology Department	Trimester of the year	1	8	7	1	57	73
		2	3	2	1	24	30
	Total		11	9	2	81	103
Total	Trimester of the year	1	855	536	319	6052	7762
		2	1136	643	498	8954	11,231
	Total		1991	1179	817	15,006	18,993

MDR—multidrug resistant; ESBL—extended-spectrum beta-lactamase; KESC—*Klebsiella* spp., *Enterobacter* spp., *Serratia* spp., and *Citrobacter* spp.; AB-MDR—*Acinetobacter baumanii* multidrug resistant; VRE—vancomycin-resistant *Enterococcus*; MRSA—methicillin-resistant *Staphylococcus aureus*.

**Table A3:** Crosstabulation between screening results by type of swab.

		Type of Swab				Total
		Nasal Swab	Axillary Swabs	Inguinal Swabs	Rectal/Perianal Swabs	
Screening Result	Axillary Swabs-screening MRSA-positive	0	55	0	0	55
	Axillary Swabs-screening MRSA-negative	0	1124	0	0	1124
	Inguinal Swab-screening MRSA-positive	0	0	75	0	75
	Inguinal swabs-screening MRSA-negative	0	0	742	0	742
	Proteeae group bacteria-MDR-positive	0	0	0	33	33
	KESC bacteria group-MDR-negative	0	0	0	3435	3435
	KESC bacteria group-MDR-positive	0	0	0	491	491
	Proteeae group bacteria-MDR-negative	0	0	0	1931	1931
	Nasal Swab-screening MRSA-positive	141	0	0	0	141
	Nasal Swab-screening MRSA-negative	1850	0	0	0	1850
	*E. faecium*-VRE-positive	0	0	0	157	157
	*E. faecium*-VRE-negative	0	0	0	1806	1806
	*E. faecalis*-positive	0	0	0	4	4
	*E. faecalis*-negative	0	0	0	1959	1959
	*E. coli*-ESBL-positive	0	0	0	342	342
	*E. coli*-ESBL-negative	0	0	0	3584	3584
	AB-MDR-positive	0	0	0	62	62
	AB-MDR-negative	0	0	0	1202	1202
Total		1991	1179	817	15,006	18,993

MDR—multidrug resistant; ESBL—extended-spectrum beta-lactamase; KESC—*Klebsiella* spp., *Enterobacter* spp., *Serratia* spp., and *Citrobacter* spp.; AB-MDR—*Acinetobacter baumanii* multidrug resistant; VRE—vancomycin-resistant *Enterococcus*; MRSA—methicillin-resistant *Staphylococcus aureus*. Out of 1991 nasal swabs screened, 141 tested positive for MRSA, while 1850 were negative. Among 1179 axillary swabs, 55 were positive for MRSA, and 1124 were negative. For inguinal swabs, a total of 817 samples were collected, with 75 testing positive for MRSA and 742 negative. The most extensive range of screenings occurred in the rectal/perianal swab category, with a total of 15,006 samples. In this group, 3435 samples were MDR-negative, and 491 were MDR-positive for the KESC bacteria group, while the Proteeae group had 1931 MDR-negative and 33 MDR-positive cases. For *E. faecium*, 1806 samples were VRE-negative, and 157 were VRE-positive. In the *E. coli*-ESBL screening, 3584 were ESBL-negative, and 342 were ESBL-positive. *E. faecalis* was detected in 1959 with negative cases and only 4 positive cases, while for AB-MDR, 1202 samples were negative, and 62 tested positive for multidrug resistance.

**Table A4:** Crosstabulation table of hospitalization department by screening results.

Count		AB-MDR-Negative	AB-MDR-Positive	*E. coli*-Negative	*E. coli*-Positive	*E. faecalis*-Negative	*E. faecalis*-Positive	*E. faecium*-Negative	*E. faecium*-Positive	Nasal Swab-Screening Methicillin-Resistant *Staphylococcus aureus*-Negative	Nasal Swab-Screening Methicillin-Resistant *Staphylococcus aureus*-Positive	Proteeae Group Bacteria-MDR-Negative	KESC Bacteria Group-MDR–Positive	KESC Bacteria Group-MDR-Negative	Proteeae Group Bacteria-MDR-Positive	Inguinal Swab-Screening Methicillin-Resistant *Staphylococcus aureus*-Negative	Inguinal Swab-Screening Methicillin-Resistant *Staphylococcus aureus*-Positive	Axillary Swab-Screening Methicillin-Resistant *Staphylococcus aureus*-Negative	Axillary Swab-Screening Methicillin-Resistant *Staphylococcus aureus*-Positive	Total
Department	Allergology Department	4	0	17	5	11	0	8	3	11	0	11	0	22	0	2	0	9	0	103
	1st Anesthesia and Intensive Care Unit Clinic	368	15	1210	96	651	2	614	39	591	62	644	180	1126	9	7	2	609	36	6261
	Anesthesia and Intensive Care Unit Dedicated to Neurosurgical, Plastic Surgery, and Other Surgical Departments	83	26	312	32	171	0	145	26	150	21	168	92	252	4	122	30	17	2	1653
	Infectious Diseases Clinic	101	8	311	49	179	1	165	15	168	12	176	43	317	4	160	14	5	1	1729
	1st Cardiology Clinic	7	0	19	1	10	0	10	0	9	1	10	1	19	0	3	0	7	0	97
	2nd Cardiology Clinic	4	0	17	3	10	0	9	1	9	1	10	2	18	0	4	0	5	1	94
	1st Surgical Clinic—Thoracic Surgery Department	6	0	11	3	7	0	5	2	7	0	5	7	7	2	0	0	7	0	69
	2nd Surgical Clinic—Vascular Surgery Department	4	0	14	0	7	0	6	1	5	2	7	3	11	0	0	0	7	0	67
	1st Surgical Clinic	1	0	4	0	2	0	2	0	2	0	2	1	3	0	1	0	1	0	19
	2nd Surgical Clinic	5	0	17	1	9	0	6	3	7	2	9	7	11	0	0	0	9	0	86
	Diabetes Clinic	0	0	2	0	1	0	0	1	1	0	1	2	0	0	1	0	0	0	9
	Gastroenterology Clinic	6	0	15	1	8	0	8	0	8	0	8	5	11	0	0	0	7	1	78
	Haematology Clinic	23	0	50	6	28	0	23	5	28	0	28	10	46	0	0	0	28	0	275
	1st Internal Medicine Clinic	189	5	586	58	322	1	300	23	302	20	317	38	606	6	8	0	302	12	3095
	2nd Internal Medicine Clinic	179	1	472	48	260	0	239	21	246	14	255	51	469	5	236	23	0	1	2520
	Neurosurgical Clinic	3	0	15	1	8	0	6	2	8	0	8	2	14	0	4	0	4	0	75
	Neurology Clinic	24	2	47	5	26	0	26	0	24	2	25	13	39	1	8	2	15	1	260
	Obstetrics and Gynecology Clinic	1	0	2	0	1	0	1	0	1	0	1	0	2	0	1	0	0	0	10
	Oncology Clinic	3	0	8	2	5	0	5	0	5	0	5	0	10	0	2	0	3	0	48
	Orthopedics and Traumatology Clinic	47	0	128	10	69	0	65	4	97	2	69	9	129	0	86	3	12	0	730
	1st Medical Rehabilitation Clinic	6	0	14	0	7	0	7	0	7	0	6	0	14	1	4	0	4	0	70
	Medical Rehabilitation Clinic—Neurological Department	39	3	88	4	46	0	44	2	45	0	45	4	88	1	24	0	22	0	455
	Medical Rehabilitation Clinic—Orthopedics and Traumatology Department	25	0	51	3	27	0	27	0	27	0	27	0	54	0	18	0	9	0	268
	Rheumatology Clinic	16	0	36	6	21	0	20	1	21	0	21	4	38	0	0	0	21	0	205
	Cardiology Intensive Care Department	49	1	111	7	59	0	52	7	57	2	59	13	105	0	46	1	12	0	581
	Urology Clinic	5	0	18	0	9	0	9	0	9	0	9	0	18	0	4	0	5	0	86
	2nd Anesthesia and Intensive Care Unit Clinic	2	0	4	0	2	0	2	0	2	0	2	0	4	0	0	0	2	0	20
	Nephrology and Dialysis Clinic	2	1	5	1	3	0	2	1	3	0	3	4	2	0	1	0	2	0	30
Total		1202	62	3584	342	1959	4	1806	157	1850	141	1931	491	3435	33	742	75	1124	55	18,993

MDR—multidrug resistant; ESBL—extended-spectrum beta-lactamase; KESC—*Klebsiella* spp., *Enterobacter* spp., *Serratia* spp., and *Citrobacter* spp.; AB-MDR—*Acinetobacter baumanii* multidrug resistant; VRE—vancomycin-resistant *Enterococcus*; MRSA—methicillin-resistant *Staphylococcus aureus*.
